# A Supply and Demand Management Perspective on the Accelerated Global Introductions of Inactivated Poliovirus Vaccine in a Constrained Supply Market

**DOI:** 10.1093/infdis/jiw550

**Published:** 2017-06-30

**Authors:** Ian Lewis, Ann Ottosen, Jennifer Rubin, Diana Chang Blanc, Simona Zipursky, Emily Wootton

**Affiliations:** 1 UNICEF Supply Division, Copenhagen, Denmark;; 2 World Health Organization, Geneva, Switzerland;; 3 Gavi, the Vaccine Alliance, Geneva, Switzerland (until September 2015)

**Keywords:** IPV introductions, IPV shortage.

## Abstract

A total of 105 countries have introduced IPV as of September 2016 of which 85 have procured the vaccine through UNICEF. The Global Eradication and Endgame Strategic Plan 2013-2018 called for the rapid introduction of at least one dose of IPV into routine immunization schedules in 126 all OPV-using countries by the end of 2015. At the time of initiating the procurement process, demand was estimated based on global modeling rather than individual country indications. In its capacity as procurement agency for the Global Polio Eradication Initiative and Gavi, the Vaccine Alliance, UNICEF set out to secure access to IPV supply for around 100 countries. Based on offers received, sufficient supply was awarded to two manufacturers to meet projected routine requirements. However, due to technical issues scaling up vaccine production and an unforecasted demand for IPV use in campaigns to interrupt wild polio virus and to control type 2 vaccine derived polio virus outbreaks, IPV supplies are severely constrained. Activities to stretch supplies and to suppress demand have been ongoing since 2014, including delaying IPV introduction in countries where risks of type 2 reintroduction are lower, implementing the multi-dose vial policy, and encouraging the use of fractional dose delivered intradermally. Despite these efforts, there is still insufficient IPV supply to meet demand. The impact of the supply situation on IPV introduction timelines in countries are the focus of this article, and based on lessons learned with the IPV introductions, it is recommended for future health programs with accelerated scale up of programs, to take a cautious approach on supply commitments, putting in place clear allocation criteria in case of shortages or delays and establishing a communication strategy vis a vis beneficiaries.

In May 2013 the World Health Assembly endorsed the new Polio Eradication and Endgame Strategic Plan 2013–2018 [1], which under objective 2 calls for all countries to strengthen routine immunization programs and replace trivalent oral poliovirus vaccine (OPV) with bivalent OPV by 2016. To minimize the risks associated with oral poliovirus vaccine type 2 (OPV2) withdrawal, the Strategic Advisory Group of Experts on Immunization (SAGE) had already recommended [2] the introduction of ≥1 dose of inactivated poliovirus vaccine (IPV) into all routine immunization programs in November 2012 and indicated its intent to review progress, including toward the availability of affordable IPV products. In April 2014 SAGE concurred, based on the vaccine prices achieved, that the prices obtained constituted a firm basis for proceeding with the Polio Eradication and Endgame Strategic Plan 2013–2018 goal of introducing ≥1 dose of IPV by the end of 2015 in all countries that had been using OPV only [3].

Although the IPV had been in use since 1955, by January 2013 the vaccine was only part of the national immunization schedule in 65 countries. These countries had either IPV-only schedules (a full 3-dose IPV schedule) or used IPV as part of a sequential OPV/IPV schedule, and generally administered IPV in a combination vaccine. This meant that 126 “OPV-only” countries would be required to introduce IPV within 17 months from the reconfirmation by SAGE in April 2014 to meet the PEEPS timeline. Achieving this target would require the vaccine introduction to proceed at an unprecedented rate, compared with any previous vaccine introductions in history. From a vaccine supply perspective, this provided several challenges. First, the scale-up in demand over a short period of time would require an increase in annual global supply, from about 80 million doses in 2013 to about 190 million by 2016 [4]. Second, significant quantities of IPV as a stand-alone presentation would now be required, not just in a single-dose but also in multidose vials. This article explores, from a vaccine supply perspective, the experience of IPV introduction through September 2016, to identify any lessons to be learned for future global health initiatives that may be rolled out on a similarly ambitious scale.

## LEADING UP TO THE GLOBAL IPV INTRODUCTION POLICY RECOMMENDATION

In 2013, manufacturers produced about 80 million doses of IPV annually in different formulations, primarily in pentavalent or hexavalent combination vaccines and with some supply of IPV as a stand-alone vaccine in prefilled syringes or vials. The market was primarily high-income self-procuring countries, and therefore demand through the United Nations Children’s Fund (UNICEF) was limited, amounting to about 300 000–500 000 doses annually for 2–5 countries between 2004 to 2013. As early as 2012, the Global Polio Eradication Initiative (GPEI) was considering using IPV in a campaign setting in key endemic countries, with a projected requirement of up to 23 million doses. UNICEF, in its capacity as procurement agency for the GPEI, undertook a procurement process to meet the projected requirement, but though manufacturers offered a sufficient supply, the programmatic uptake for campaigns did not materialize. The outcome of the tender—the procurement process through which UNICEF as a public buyer issues and processes solicitation documents to ensure competition—was an award to 1 supplier for routine immunization requirements (300 000 doses).

Although the results were disappointing to the manufacturers, the tender did provide some useful information around IPV pricing and manufacturing capacity for future IPV use, with price indications between $2.00 and $5.70 per dose [5], and indications that supply was sufficient to meet demand. Before the issuance of UNICEF’s IPV tender in October 2013, the GPEI had received assurances from industry on the current and future capacity for IPV, with an understanding that a scale-up at the level required for introductions by the end of 2015 would be feasible. Indeed, during the planning of the IPV projections and the implementation of the IPV tender, the main concerns of the GPEI and manufacturers were related to demand and whether countries would be willing and able to introduce IPV within the required timeline, considering competing priorities. 

**Figure 1.**
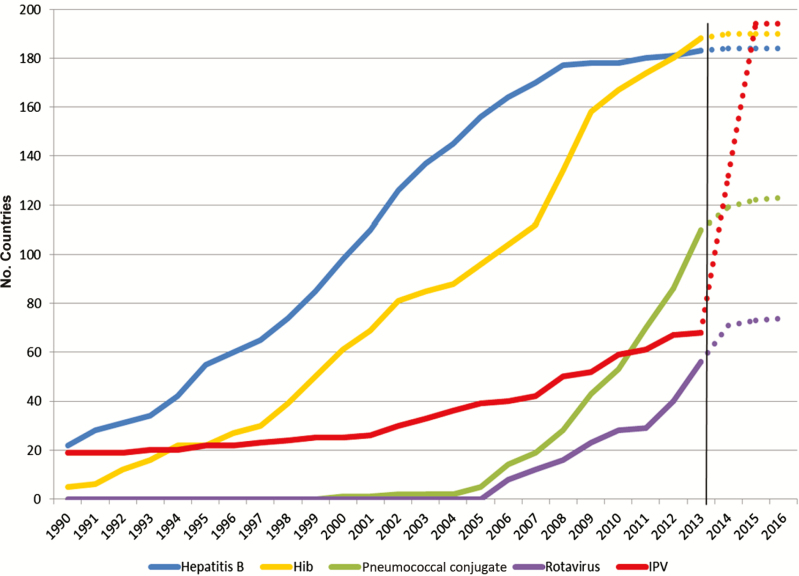
Rate of introduction of new vaccines in all countries, 1990–2013.

The experiences with new vaccine introductions since 2001 in the 73 countries supported by Gavi illustrated that, despite access to vaccines and funding, new vaccine introductions often rolled out slower than projected by partners. This was experienced with the pentavalent vaccine, which was introduced in the first Gavi-supported country in 2001 and the last country in 2015. Pneumococcal conjugate vaccine has been the fastest rollout for a Gavi-supported vaccine with 55 of 73 countries having introduced the vaccine as of September 2016, 6–7 years after the vaccines first became prequalified by the World Health Organization (WHO). To meet the GPEI timelines, IPV introductions would have to be much faster (Figure 1).

## PROJECTING DEMAND AND SECURING SUPPLY WITHOUT CONFIRMED DEMAND

In October 2013, UNICEF issued a tender covering forecasted demand for the period 2014 to 2018. When developing the demand forecast for the second and third quarters of 2013, countries had not yet started planning for IPV introductions, so no indication of planned introduction dates requirements were available to guide the partners. The methods were was based on the Gavi Strategic Demand Forecast and further adapted to meet the GPEI end-game strategy assumptions and requirements, including projections of introduction dates based on countries’ past experience with the introduction of other new vaccines, as well as a programmatic priority for countries required for earlier introduction; country-level birth cohorts; routine immunization coverage levels based on WHO and UNICEF estimates of national immunization coverage (WHO and UNICEF Estimates of National Immunization Coverage estimates); wastage rates for 1-, 5-, and 10-dose vial presentations; 1 dose per schedule; and assumption of in-country strategy for rollout (country wide or phased). 

IPV-introducing countries had been categorised on a risk-tier basis in line with the GPEI tiering scheme: the endemic countries and countries at highest risk of re-importation of WPV, and countries with outbreaks of type 2 vaccine derived polio viruses (VDVPs) associated with low immunisation coverage (<80%), were classified as Tier 1 and Tier 2 countries respectively; and the remaining countries scheduled for IPV introduction fell under the Tier 3 and the lowest risk Tier 4 category. In its tender, UNICEF included demand for 404 million doses for the period from 2014 to 2018, to cover (1) all Gavi-supported countries, including India and Indonesia (except Pan American Health Organization [PAHO] countries, whose vaccine requirements were included under a separate PAHO tender), and (2) middle-income countries that either at that time procured or could potentially consider procuring IPV through UNICEF (including China).

At the time the tender was issued, 1-, 2-, and 10-dose vial presentations were prequalified by WHO from 4 vaccine manufacturers, and a 5-dose vial was in development. However, owing to the considerably higher wastage rates of the 10-dose vials (estimated at 50%), the nonapplicability of the multidose-vial policy for the IPV at that time, the higher cold-chain requirements for the single-dose vial and the anticipation that the price would be high for single-dose vials, the GPEI indicated preferences for a smaller multidose-vial presentation, such as a 5-dose vial. The tender therefore requested 1-, 5-, and 10-dose vials but allowed manufacturers to submit proposals for any presentation. Although the expectation from partners was that countries would prefer 5-dose vials, countries’ preferred presentations were eventually found to be evenly distributed between the 1-, 5-, and 10-dose vials, owing to programmatic differences between countries. Single-dose presentations are preferred in high-income countries, but the majority of vaccines procured through UNICEF are in multidose-vial presentations. The preferred vial size is a function of the country context, including birth cohort, immunization schedule, service delivery modality, vaccination strategy, price per dose, wastage acceptance, cold-chain capacity, and applicability of the multidose-vial policy; thus, there are no global policies in this area.

The tender was issued to all 4 manufacturers that produced a WHO-prequalified IPV, as well as all manufacturers known by UNICEF and partners to have an IPV product in development. Offers for IPVs were received from all manufacturers with WHO-prequalified vaccines as well as several manufacturers with vaccines in development; however, only 2 of 4 manufacturers with prequalified vaccines offered substantial quantities for delivery during the period of 2014–2018. The other 2 manufacturers of prequalified vaccines offered only very limited quantities.

**Figure 2.**
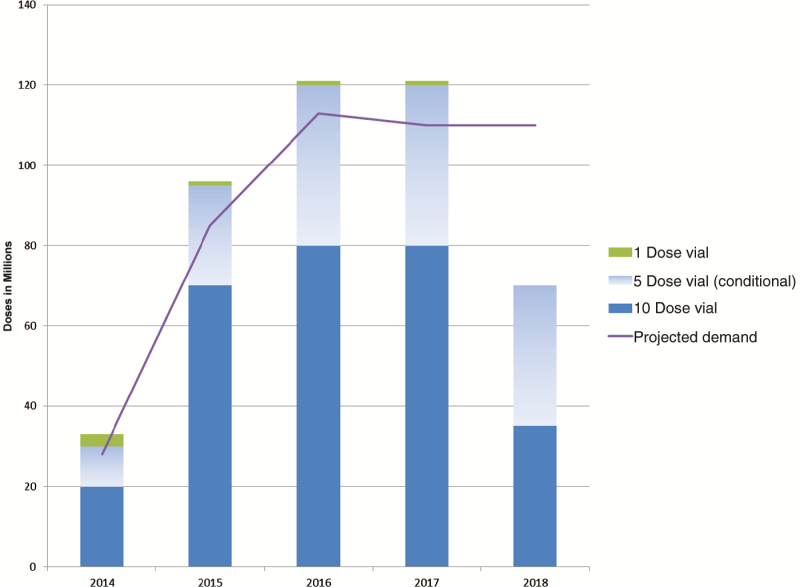
Projected demand at time of awards and awarded supply, 2014–2018.

Total awards of 441 million doses were made in February 2014, ensuring sufficient supply to meet the projected demand, while not fully awarding requirements for 2018. The purpose of leaving quantities unawarded was to ensure market incentives for pipeline manufacturers to accelerate development, because they had indicated that supply would potentially become available by 2017–2018 (Figure 2).

The IPV prices were shared with SAGE, which concurred that these represented the best possible IPV prices in the near term and constituted a firm basis for proceeding with the goal of IPV introduction by the end of 2015 [6]. For Gavi-supported countries the prices ranged from €0.75 per dose (approximately $1.00 per dose at the exchange rate at the time) in a 10-dose vial to $2.80 per dose in a single-dose vial. For middle-income countries the prices ranged from €1.50 to €2.40 per dose in a 10-dose vial (approximately $2.10–3.30 per dose at the exchange rate at the time) and $2.80 per dose in a single-dose vial. Since the confirmation from SAGE, the 5-dose vial was prequalified in November 2014, being available to all countries at a price of $1.90 per dose. With supply commitments above projected demand, the Immunization Management Group (IMG) could therefore focus efforts on working with countries to stimulate demand and clarify presentation preferences, to ensure that demand would pick up at a similar pace as the supply.

## PLANS VERSUS REALITY FOR IPV SCALE-UP

Despite the reassurances to the GPEI and UNICEF before the tender and during the award phase that supply would be available, both manufacturers experienced significant delays as they scaled up production. This resulted in a reduction of 216 million doses of IPV from the initial awards of 441 million doses, as of September 2016. This meant that the actual amount of IPV available for the 2014–2018 period is projected to reach only about 51% of awarded quantities. The impact of the reduction in available supply was significant, because the GPEI was fully relying on supply commitments to meet the aggressive requirements for country introductions to be completed by the end of 2015. 

**Figure 3.**
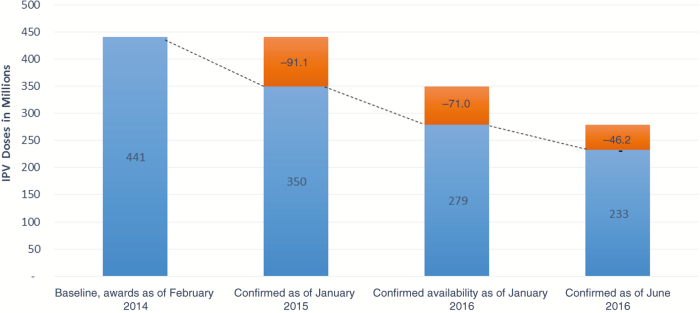
Cumulative supply reductions across suppliers, February 2014 to June 2016.

The reduced availability from manufacturers was due to many factors, but chief among them was overoptimistic planning from both manufacturers, which in turn led to a delayed ability to produce bulk vaccine at the scale required. Other factors influencing the reduced availability of IPV included unplanned production stops, delays in restarting production after maintenance, delays due to installation and validation of new equipment, breakdown of equipment, delays in licensure of the 5-dose vial, delays in releases due to implementation of new systems, technical issues with the 1-dose vial, and lack of buffer stocks at all levels of production, causing any reduction or delay to directly affect supply availability. Since 2014, the realization of the inability to meet commitments from manufacturers happened gradually, and it is considered likely that further reductions in supply may materialize in 2017 and 2018 (Figure 3).

## MATERIALIZATION OF DEMAND FOR IPV

Despite initial reservations, strong demand for IPV for the routine immunization program did materialize. In fact, all 126 countries that had been using only OPV have committed to introducing 1 dose of IPV into their routine immunization programs. However, as of September 2016, only 105 of 126 countries required have introduced IPV owing to supply constraints. At the time of estimating the demand for the UNICEF tender, the GPEI did not consider that IPV would be required for campaigns or outbreak response. However, after the awards were made to manufacturers, the GPEI reconsidered the utility of IPV for these strategies and decided to apply IPV in select campaigns as part of the outbreak response as well as in specific areas in endemic countries to support eradication, which further increased demand. In total, 18 million doses—corresponding to 15%—of the total available supply projected through to the end of 2016 have been allocated for these activities.

## MULTIPRONGED APPROACH FOR MITIGATING SUPPLY SHORTAGES

UNICEF and GPEI partners have been working closely with manufacturers to monitor the IPV supply situation, through weekly calls, bimonthly face-to-face meetings, and ad hoc high-level meetings as supply allocations and deliveries at the operational level required increasingly careful management. To make the best use of the constrained supply, close monitoring and management of IPV stocks at country and global level were implemented. This resulted in increased logistic costs and workload, because deliveries for routine requirements were released only on receipt of information from countries on available stocks, only limited quantities were delivered each time, thus requiring multiple shipments, and only partial delivery of the standard buffer stocks of 3-month supplies was provided. This tight supply management increased the risks of country stockouts but avoided overstocking in any country and ensured that more countries could have access to vaccines. In an attempt to bridge the supply gap, UNICEF and GPEI partners also followed up with the other 2 manufacturers with a prequalified IPV, but without success. A tender for procurement of vaccines licensed in India, but not prequalified by WHO, was issued to ensure access to locally available supply to meet the requirements of India.

In autumn 2014, the latest information from manufacturers indicated that supply would not be sufficient to meet demand, and it became necessary to introduce criteria to allocate the available supply between the 126 OPV-only countries, outbreak response efforts, and the endemic countries. In October 2014, the Polio Oversight Board (POB) endorsed a proposal from the IMG supply group to prioritize available IPV supply as follows: (1) Planned campaigns in endemic countries within an agreed cap; (2) routine introductions in tier 1 and 2 countries; (3) routine introductions in tier 3 and 4 countries; and (4) additional unplanned campaigns in endemic countries and in nonendemic countries.

As the supply gap increased over the following years, and as new data became available on the impact of using IPV in outbreak response, further prioritization was required. In April 2016 the POB reconfirmed the use of IPV to interrupt wild poliovirus transmission as its first priority, with routine requirements for tier 1 and 2 countries as the second priority. However, owing to further reductions in supply, it became apparent that not all countries would be able to introduce before the switch and that supply would therefore be required as a third priority for outbreak response to type 2 VDPV after the switch. The prioritization scheme also aimed to provide clarity to tier 3 and 4 countries—which would need to have their IPV introductions delayed—as to when they could expect IPV, so they could adjust their plans accordingly.

The GPEI also led several technical efforts to increase supply availability by stretching the available doses. First, based on a review of new preservative efficacy data from studies of 2-phenoxyethanol conducted by the 2 manufacturers, validated by the national regulatory authorities, and in line with the European pharmacopeia guidelines, in November 2014 WHO’s prequalification team confirmed that the data supported the use of opened multidose vials of IPV in subsequent sessions [7]. This allowed IPV to be kept at the healthcare facilities for routine immunization when appropriately stored in the cold chain for up to 28 days after opening—instead of having to be discarded after 6 hours—and thereby theoretically reduced the estimated wastage from 50% to 20% for 10-dose and from 30% to 15% for 5-dose vials. Although the multidose-vial policy is not applied in all countries or in all settings (eg, not for routine immunizations through outreach where routine immunizations are provided in the community and vaccines are discarded at the end of the activity), this new policy has stretched the supply of IPV and allowed additional countries to introduce it.

Second, several studies [8, 9] showed that immunogenicity of 2 fractional doses (0.1 mL) administered intradermally is superior to that of 1 full dose (0.5 mL) administered intramuscularly. This evidence led SAGE in April 2016 to encourage countries to evaluate the costs and benefits, trade-offs, and programmatic feasibility associated with introduction of a 2-dose fractional schedule, for example, at 6 and 14 weeks instead of a single full dose at 14 weeks [10]. So far, fractional dosage is being applied in the routine immunization program in 16 states in India. This served as a model for Sri Lanka, which also introduced fractional dosage for its routine immunization program in July 2016. With this strategy, India has ensured sufficient supply to immunize all children, and application of fractional dosing in Sri Lanka will allow the available supply in the country to ensure ongoing vaccinations through mid-2017 instead of stocking out in the last quarter of 2016. Furthermore, IPV in fractional doses has been used in a small response to a type 2 event in India and is planned for use in Pakistan. These activities have therefore increased the number of children being immunized as part of the routine immunization program, as well as stretching available supply for outbreaks, in line with the prioritization determined by the POB. Although further efforts to expand the use of fractional dosing of IPV are required as recommended by SAGE, in October 2016, barriers at the country level are that this administration form and dosage are not part of the licensure (ie, they are “off label”) and countries therefore need to take responsibility; that 2 doses are required instead of 1; and that in many countries healthcare workers are not trained in giving injections intradermally.

Despite these efforts, the available supply of IPV was still not sufficient and resulted in the undesirable situation of having to stop resupply of routine vaccines to countries that had already introduced IPV. This is expected to lead to stockouts and a temporary halt to the IPV routine program in about 23 countries, with another 20 countries required to delay IPV introduction until the fourth quarter of 2017. These countries are all tier 3 and 4 countries, considered at relatively lower risk of type 2 reintroduction, in line with the criteria established by the POB. Reductions in available supply are communicated by manufacturers as they experience unexpected challenges, at which point affected countries are informed of changes to their IPV supply plan. This means that many of these countries have been informed about the need to delay introductions several times. Although efforts were undertaken by partners to ensure the coordination of clear and consistent messages on changes in supply and the expected impact on a country-by-country basis, the lack of overall clarity and the piecemeal provision of information on availability have been frustrating for countries, particular after the initial strong recommendation from the GPEI for countries to introduce IPV by the end of 2015. This has unfortunately led to a loss of confidence in the program in some countries.

## LESSONS LEARNED

Several lessons can be learned from this experience of a globally planned introduction of a vaccine in a high number of countries in a short period of time. First, the time required to scale up vaccine production of even a well-known vaccine is often underestimated. Second, establishing allocation criteria from the onset based on programmatic requirements is critical. Finally, clear, coordinated, and timely communication to countries on vaccine availability issues that may impact their plans is essential.

### Challenges of Scaling Up Vaccine Production

Based on the planned scaling up of production capacity as assessed by the 2 awarded manufacturers in November 2013, vaccine demand could be fully met throughout 2014–2018. In reality, however, supply has yet to reach the annual awarded quantities from either of the 2 manufacturers. Based on supply reductions as of September 2016, it is anticipated that only about 51% of awarded quantities will be made available. There have been multiple notifications about reductions in annual availability as well as delays in timing of availability of IPV from both manufacturers, with a high probability of further reductions in the future. Should this happen, it will require further reductions in IPV activities in accordance with the criteria established by the POB, which would probably change the timing of the availability of the outbreak stock pile as well as deliveries for tier 2 countries. The manufacturer with the largest capacity for IPV production, which is producing other vaccines at large scale, has indicated that it does not expect to reach full-scale production for IPV until 2019, several years after its initial projections. 

The other manufacturer with an initial production capacity estimated at 20 million doses of bulk annually has yet to produce at this scale. To increase the capacity, this manufacturer planned improvement to both upstream and downstream processes as well as the commissioning of a new facility. The changes to the processes did not fully meet the expected yield improvements, and the new facility originally planned to be functional in 2017 is likely to be delayed. Although both manufacturers have been supportive of dose stretching when it came to studies required for application of the multidose-vial policy, there has been no willingness to pursue licensure for fractional dose application, so this application remains at country discretion and accountability as an off-label indication.

The lesson learned is that a very ambitious expansion of a vaccine program that relies on scaling up the production capacity may put the program at risk, despite the fact that production of the vaccine is well known. More manufacturers should be awarded, where possible, and access to back-up capacity should be secured to protect the program. Alternatively, the rate of acceleration for introduction could be adjusted to a more conservative timeline.

### Establishing Allocation Criteria From the Onset Based on Programmatic Requirements

If there is a risk that supply will not be sufficient to meet demand, as when production is required to ramp up to unprecedented levels within a short lead time, the early establishment of allocation criteria should be considered. This will ensure that the available supply is allocated to best meet the program priorities and that these priorities are clear from the early stages of introduction. Such criteria need to be clear, transparent, fair, and justifiable to countries that may not be prioritized for supply allocations under a constrained supply situations. These criteria should be published and shared with all countries as part of the process of planning for introduction. 

In the case of the IPV, for which prioritization criteria were not established at the outset, vaccines were allocated and delivered based on planned introductions dates including tier 3 and 4 countries, under the assumption that supply would be available. There was also a risk that supply would not be used should tier 1 and 2 countries delay introductions, considering also that many tier 3 and 4 countries were small. In retrospect, and in line with the policy for other new vaccine introductions as well as based on the updated allocation criteria established, IPV supply should not have been allocated to tier 3 and 4 countries because the uninterrupted, sustainable supply could not be guaranteed. The potential impact on other immunization programs of interrupting an ongoing routine immunization program could lead to general loss of confidence among parents, healthcare workers, and programs. It will be important to monitor any negative impact on vaccination coverage of other antigens as the stocks of IPV are coming to an end in tier 3 and 4 countries.

### Clear, Coordinated, and Timely Communication to Countries Whose Plans Could Be Altered by Vaccine Availability

In a situation with a risk to supply availability and with clear prioritization criteria established from the onset, countries that may be affected by vaccine shortages should be fully informed to ensure that they understand the uncertainties and the potential risks. They could then be given the option to wait until there is certainty that the sustainable and uninterrupted supply can be secured for vaccine introduction or introduce the vaccine earlier, knowing the potential risks of a stockout. In the case of IPV, many countries were notified several times about changes in the timing of when they would receive their IPV for introduction, owing to frequent reductions in forecasted availability from the manufacturers, requiring them to interrupt or restart preparatory activities with short notice.

In conclusion, the aggressive goal for 126 OPV-only countries to introduce IPV before the end of 2015 was not achieved. As of September 2016, IPV has been introduced in 105 of these countries, a testament to the commitment of the countries and partners to work together to achieve such an ambitious target. Although there were reservations about the extent to which demand would materialize, the limiting factor for achieving the goal turned out to be the insufficient supply of IPV, owing to challenges in scaling up a well-known vaccine, and this experience therefore provides important lessons learned for similar initiatives that may arise in the future.

## Notes

***Supplement sponsorship.*** This work is part of a supplement coordinated by the Task Force for Global Health with funding provided by The Bill and Melinda Gates Foundation and the Centers for Disease Control and Prevention.

***Potential conflicts of interests.*** All authors: No reported conflicts. All authors have submitted the ICMJE Form for Potential Conflicts of Interest. Conflicts that the editors consider relevant to the content of the manuscript have been disclosed.
